# Efficacy of Forward and Reverse Suturing Techniques in Enhancing Neural Regeneration and Motor Function Recovery Following Facial Nerve Axotomy

**DOI:** 10.3390/jcm15010096

**Published:** 2025-12-23

**Authors:** Jae Min Lee, Yeon Ju Oh, Sung Soo Kim, Youn-Jung Kim, Seung Geun Yeo

**Affiliations:** 1 Department of Otorhinolaryngology—Head and Neck Surgery, School of Medicine, Kyung Hee University Medical Center, Seoul 02447, Republic of Korea; jmlee3042@khu.ac.kr; 2 College of Nursing Science, Kyung Hee University, Seoul 02447, Republic of Korea; yj129@khu.ac.kr; 3 Department of Medicine, College of Medicine, Kyung Hee University Medical Center, Seoul 02447, Republic of Korea; 5duswn1203@khu.ac.kr; 4 Department of Biochemistry and Molecular Biology, College of Medicine, Kyung Hee University, Seoul 02447, Republic of Korea; sgskim@khu.ac.kr

**Keywords:** facial nerve injury, axotomy, motor recovery, forward suturing, reverse suturing

## Abstract

**Background/Objectives**: Facial nerve injury from conditions such as Bell’s palsy, trauma, surgery, and infection leads to facial asymmetry and motor deficits. Axotomy models reproduce peripheral nerve disruption and consequent motor impairment. To compare the effects of forward versus reverse autologous nerve suturing on neural regeneration and motor recovery within the facial nucleus after axotomy. **Methods**: In rats subjected to facial nerve axotomy, motor recovery was assessed at 8 weeks using whisker movement and blink reflex tests. Immunohistochemistry quantified choline acetyltransferase (ChAT), sirtuin 1 (SIRT1), and Iba-1 as indices of cholinergic function, cellular stress/inflammation modulation, and microglial activation in the facial nucleus. **Results**: Axotomy significantly reduced whisker and blink scores compared with sham. Both forward and reverse suturing significantly improved these behavioral outcomes versus axotomy. Within the facial nucleus, axotomy decreased ChAT- and SIRT1-positive cells and increased Iba-1 expression, while both suturing techniques increased ChAT and SIRT1 and reduced Iba-1. These changes suggest enhanced cholinergic function, mitigation of stress/inflammatory responses, and attenuation of microglial activation following repair. **Conclusions**: Forward and reverse suturing were each associated with improved motor function and favorable molecular and cellular changes in the facial nucleus after facial nerve axotomy. These findings support the utility of surgical repair irrespective of graft orientation and highlight involvement of key pathways—cholinergic signaling, SIRT1-related regulation, and microglial activity—in nerve restoration. This work extends our previous study, which focused on peripheral nerve regeneration after forward and reverse suturing, by elucidating how graft orientation affects central facial nucleus responses. By integrating behavioral outcomes with ChAT, Iba-1, and SIRT1 expression, the present study provides novel insight into the central mechanisms underlying motor recovery after facial nerve repair and helps explain why comparable functional outcomes are achieved regardless of graft polarity.

## 1. Introduction

Facial nerve injury involving the seventh cranial nerve leads to weakness or paralysis of the facial muscles, resulting in facial asymmetry and impaired functions such as eye closure, smiling, and grimacing [1]. Such injuries can arise from idiopathic palsy, infections, neoplastic lesions, or trauma to the skull and face. When the facial nerve is transected, surgical reconstruction is required to restore function; depending on the defect length, this may involve direct end-to-end neurorrhaphy or the use of interposition nerve grafts to bridge the gap and facilitate axonal regeneration [2].

In animal models of facial nerve injury, axotomy—complete transection of the facial nerve—is widely used to induce severe and long-lasting deficits that are often not fully reversible, even after surgical repair. When a nerve gap prevents direct end-to-end suturing, autologous nerve grafts are interposed to create a regenerative pathway that facilitates axonal regrowth [3]. In this context, the orientation of the autologous graft has been considered a critical factor: one approach aligns the graft in the same proximal-to-distal direction as the original nerve (forward suturing), whereas the other reverses this orientation (reverse suturing), thereby inverting the longitudinal alignment of nerve fibers [4]. Traditionally, it has been assumed that maintaining the original polarity of nerve grafts provides a more natural guidance pathway and thus improves regeneration, but several experimental studies have reported that reversing graft orientation does not significantly alter axonal regeneration or functional outcomes, suggesting that graft function may be relatively independent of its polarity [5]. These contrasting findings highlight the complexity of axonal regeneration and indicate that factors beyond simple anatomical alignment may influence the success of peripheral nerve repair. Our previous research further supports these findings: using the same facial nerve axotomy and autograft model, we showed that forward and reverse suturing produced comparable peripheral nerve regeneration and functional recovery, with no significant differences in microstructural outcomes between the two orientations [6]. However, that work focused exclusively on the peripheral nerve and did not address how graft orientation affects central adaptations within the facial nucleus. Given that facial motor recovery depends not only on peripheral axonal regrowth but also on plastic changes in central motor circuits, it is essential to elucidate whether forward and reverse suturing differentially modulate central neuronal and microglial responses.

Following peripheral facial nerve injury, marked secondary changes occur within the facial nucleus of the central nervous system, affecting both motoneurons and microglia [7]. Injured motor neurons undergo degeneration and synaptic loss, leading to impaired neuronal activity. Choline acetyltransferase (ChAT), the key enzyme for acetylcholine synthesis, is essential for cholinergic motor neuron function, and its expression level in the facial nucleus is widely used as an indicator of motoneuronal integrity and functional status after injury [7]. In parallel, microglial cells become activated and proliferate around injured motoneurons, releasing inflammatory mediators that can exacerbate neuroinflammation and contribute to motor neuron degeneration. Ionized calcium-binding adaptor molecule 1 (Iba-1) serves as a marker of activated microglia, and changes in Iba-1 expression reflect the degree of microglial activation and neuroinflammatory status [8]. Facial nerve injury also induces oxidative stress. Sirtuin 1 (SIRT1), a class III histone deacetylase, regulates multiple processes including apoptosis, inflammation, and cellular stress responses [9]. Experimental studies have shown that SIRT1 activation can enhance motor nerve regeneration by reducing oxidative stress and neuroinflammation, thereby contributing to a more pro-regenerative environment for injured motor neurons.

Facial nerve injuries can lead to significant functional impairments, often necessitating surgical interventions such as nerve suturing and grafting. However, the optimal orientation of autologous nerve grafts remains debated, and its impact on regeneration is still unclear [10]. Our previous research using the same facial nerve axotomy and autograft model showed that forward and reverse suturing produce comparable peripheral nerve regeneration and functional recovery, with no significant differences in microstructural outcomes between the two orientations [6]. That work, however, focused exclusively on the peripheral nerve and did not address how graft orientation affects central adaptations within the facial nucleus. Given that facial motor recovery depends not only on peripheral axonal regrowth but also on plastic changes in central motor circuits, it is important to clarify whether forward and reverse suturing differentially modulate central neuronal and microglial responses. Changes in the facial nucleus, including alterations in ChAT, Iba-1, and SIRT1, are known to play crucial roles in the response to facial nerve injury. Therefore, building upon our previous peripheral nerve–focused study, the present work aims to investigate the effects of forward and reverse suturing on ChAT, Iba-1, and SIRT1 expression within the facial nucleus after facial nerve axotomy and to elucidate central mechanisms that may underlie motor recovery irrespective of graft polarity.

## 2. Materials and Methods

### 2.1. Animals

Male Sprague Dawley (SD) rats, aged 6 weeks and weighing between 200 and 250 g, were sourced from Orient Bio located in Seongnam, Gyeonggi-do, Republic of Korea. The animals were kept in a controlled environment set to 22 ± 2 °C with a relative humidity of 50%. They were maintained on a 12 h light/dark cycle and had unrestricted access to food and water. Following a one-week acclimatization period, the rats were randomly divided into four experimental groups: the sham group (*n* = 6), the axotomy group (*n* = 6), the axotomy + forward suture group (*n* = 6), and the axotomy + reverse suture group (*n* = 6), Group allocation was performed using simple randomization based on a computer-generated random number sequence by an investigator who was not involved in the surgical procedures or outcome assessments. All randomized animals (*n* = 24; *n* = 6 per group) were included in behavioral and histological/immunohistochemical analyses; no experimental units or data points were excluded. Humane endpoint criteria (e.g., marked weight loss, severe distress, or infection unresponsive to treatment) were predefined, but no animals met these criteria during the study.

### 2.2. Forward and Reverse Suturing Procedures After Facial Nerve Axotomy

To perform facial nerve axotomy in rats, anesthesia was induced with 5% isoflurane (Porran, Hwaseong JW, Republic of Korea) in an 80% oxygen environment, and 3% isoflurane was maintained during the procedure. While under inhalation anesthesia, a left postauricular incision was made to expose the parotid gland and mastoid process. In the experimental group, the facial nerve trunk and its five branches (temporal, zygomatic, buccal, mandibular, and cervical) were identified. In the axotomy group, a 5 mm portion of the facial nerve trunk was completely excised. This portion was stored in 0.05 M phosphate-buffered saline (PBS), and after axotomy, the excised facial nerve trunk stored in PBS was sutured in both anterograde and retrograde directions. All neurorrhaphies were performed by the same experienced microsurgeon under an operating microscope (40× magnification). The proximal and distal coaptation sites were reconstructed using two interrupted 10-0 ETHILON™ (Black, Somerville, NJ, USA) epineurial sutures at each site, placed to match the fascicular orientation of the graft and to avoid rotational mismatch. Care was taken to approximate the nerve ends without tension or gap, and to maintain consistent alignment and suture spacing across all animals. Post-surgery, the animals were monitored for changes in weight, behavior, and wound condition for three days. In the sham group, the same postauricular incision and suturing were performed under inhalation anesthesia, but no facial nerve axotomy was conducted.

### 2.3. Behavioral Tests

To evaluate the extent of damage and recovery following facial nerve injury, assessments were conducted at 8 weeks post-injury by observing whisker movement and the eyelid blink reflex. The whisker movement test, which examines vibrissae muscle function, involved securely holding the rat and scoring whisker movement using the five-point vibrissae observation scale. A score of 5 (normal) was assigned when whiskers on the affected side moved to the front position, mirroring the movement on the uninjured side. A score of 4 indicated normal movement with a slight backward tilt, 3 for substantial movement with a backward tilt, 2 for minimal movement with whiskers laid back, and 1 for no movement with whiskers laid back. The eyelid blink reflex was assessed by delivering consistent air puffs around the eyes using an air pump. The extent of eyelid closure was rated using the Eye Closing and Blinking Reflex Observation Scale. A score of 5 denoted complete eyelid closure, 4 for 75% closure, 3 for 50% closure, 2 for contraction without full closure, and 1 for no eyelid movement. Whisker movement and eyelid blink reflex scores were assessed independently by two observers who were blinded to group allocation. The mean of their scores was used for analysis.

### 2.4. Tissue Preparation

At 8 weeks following the facial nerve injury, and after completing the behavioral tests, the rats were anesthetized using ether. Once anesthetized, the rats were perfused with 0.05 M PBS to clear the circulatory system of blood, and their brains were subsequently exposed. For immunohistochemical analyses, an additional perfusion with 4% paraformaldehyde (PFA) followed the PBS perfusion. The brains were then extracted and fixed overnight in 4% PFA at 4 °C. After fixation, the brains were dehydrated by immersion in a 30% sucrose solution at 4 °C for three days. Subsequently, the brains were serially sectioned into consecutive 40-μm coronal slices using a cryostat set to a low temperature.

### 2.5. Immunohistochemistry and Immunofluorescence

ChAT, Iba-1, and SIRT1 expression was investigated using immunohistochemistry and immunofluorescence. To inhibit non-specific antibody binding, endogenous peroxidase (HRP) activity was blocked by treating the tissues with a 3% hydrogen peroxide (H_2_O_2_) solution for 30 min, followed by three PBS washes. A blocking step was then conducted at room temperature for 2 h using a solution of 1% bovine serum albumin (BSA) combined with serum from the same species as the secondary antibody host. Post-blocking, the sections were washed with PBS three times and incubated overnight at 4 °C with primary antibodies: ChAT (Merck Millipore, Cat# AB144P, RRID:AB_2079751, goat polyclonal, IHC 1:1000, Billerica, MA, USA), Iba-1 (Abcam, Cat# ab178846, RRID:AB_2782900, rabbit monoclonal, IHC 1:500, Cambridge, UK), and SIRT1 (Abcam, Cat# ab110304, RRID:AB_10864359, mouse monoclonal, IHC 1:500, Cambridge, UK) diluted in 3% BSA and 0.3% Triton X-100. After incubation, the sections were rinsed with PBS three times and then treated with secondary antibodies at room temperature for 1 h and 30 min. Following another three PBS washes, the sections were exposed to an avidin-biotin complex (Vector Elite ABC kit, Vector Laboratories, Burlingame, CA, USA) for 1 h and 30 min. Color development was achieved using 3,3-diaminobenzidine tetrahydrochloride (DAB kit, Vector Laboratories, Burlingame, CA, USA) for 2–5 min. The tissues were mounted on slides and allowed to dry. Dehydration was performed through a graded ethanol series (70–80–90–100% for 3 min each, with 100% repeated twice), followed by clearing with xylene for 10 min. Finally, sections were covered with coverslips using Permount and examined under a light microscope (BX51, Olympus Co., Ltd., Tokyo, Japan). For quantitative analysis of immunohistochemical labeling, three coronal sections encompassing the mid-portion of the facial nucleus were analyzed per animal. Sections were selected at comparable rostrocaudal levels across animals based on anatomical landmarks. For each section, ChAT-, Iba-1-, and SIRT1-positive cells within a predefined region of interest covering the entire facial nucleus were counted manually under a light microscope at 200× magnification. Three non-overlapping fields of view were evaluated per section, and the counts were averaged to obtain one value per section. Section values were then averaged to yield a single value per animal. All counts were performed by two independent observers blinded to the experimental groups, and their counts were averaged to minimize observer bias.

For immunofluorescence, the sections were first washed with 0.05 M PBS, followed by incubation in a blocking solution containing 2% BSA and 10% normal goat serum in 0.05 M PBS for 1 h at room temperature. The sections were then incubated overnight at 4 °C with primary antibodies targeting Iba-1 (1:1000; Abcam, Cambridge, UK). Following PBS washing, the sections were treated with Alexa Fluor 594-conjugated goat anti-rabbit IgG (1:1000; Molecular Probes, Eugene, OR, USA) for 1 h at room temperature. Images were captured using a Zeiss LSM 700 confocal microscope (Oberkochen, Germany).

### 2.6. Statistical Analysis

The experimental unit was the individual rat. For each outcome, technical replicates (e.g., multiple sections or fields per animal) were averaged to yield a single value per animal, and the data therefore represent biological replicates of *n* = 6 per group. Data are presented as mean ± standard error of the mean (SEM). Statistical analyses were performed using SPSS software (version 25; IBM SPSS Corp., Armonk, NY, USA). Prior to analysis, data were inspected for normality and homogeneity of variances using the Shapiro–Wilk and Levene tests, respectively. No major violations of these assumptions were detected; therefore, group differences were assessed using one-way ANOVA, followed by Tukey post hoc tests for multiple comparisons. All randomized animals (*n* = 24; *n* = 6 per group) were included in the analyses; no experimental units or data points were excluded. A significance level of *p* < 0.05 was considered statistically significant.

## 3. Results

### 3.1. Whisker Movement and Eyelid Blink Reflex Tests Following Facial Nerve Injury

**Figure 1:**
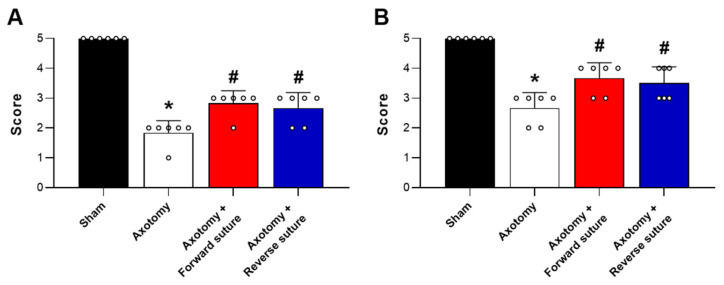
Outcomes of behavioral tests at 8 weeks post-facial nerve injury induced by axotomy. (**A**) Whisker movement (vibrissae muscle) test and (**B**) Eyelid blink reflex test. The axotomy group exhibited a significant decrease in test scores compared to the sham group, indicating impaired function due to nerve injury. In contrast, both the forward suture and reverse suture groups showed significant improvement in scores relative to the axotomy group, demonstrating the efficacy of both suturing techniques in mitigating the effects of facial nerve injury. Data are presented as means ± SEM. Statistical significance is indicated by (* *p* < 0.05 vs. Sham group) and (# *p* < 0.05 vs. Axotomy group).

The outcomes of the whisker movement (vibrissae muscle) and eyelid blink reflex tests were assessed 8 weeks after facial nerve injury induced by axotomy. In the whisker movement test, the axotomy group displayed a significant reduction in whisker reflex scores compared to the sham group, indicating impaired motor function as a result of the nerve injury. However, both the forward suture and reverse suture groups exhibited a notable improvement in whisker reflex scores compared to the axotomy group (F = 72.963, *p* < 0.001) (Figure 1A). This enhancement suggests that both suturing techniques effectively support the restoration of motor function in the vibrissae muscles. Similarly, in the eyelid blink reflex test, the axotomy group showed a significant decrease in reflex scores relative to the sham group, reflecting diminished reflexive capability due to the injury. In contrast, the forward suture and reverse suture groups demonstrated increased scores compared to the axotomy group (F = 26.867, *p* < 0.001) (Figure 1B), indicating improved recovery of the blink reflex. These results imply that suturing in either forward or reverse directions can significantly enhance functional outcomes following facial nerve injury caused by axotomy. No significant differences were detected between the forward suture and reverse suture groups for either behavioral test (*p* > 0.05).

### 3.2. Expression of ChAT in the Facial Nucleus Following Axotomy and Nerve Repair

**Figure 2:**
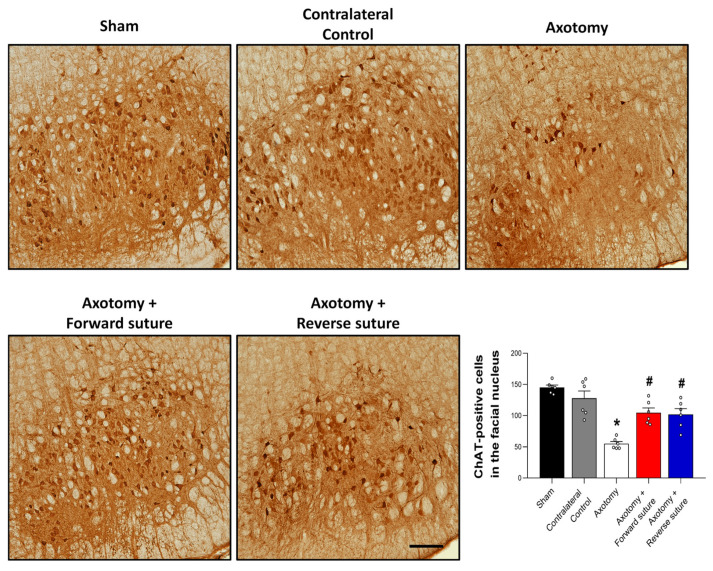
Expression of choline acetyltransferase (ChAT) in the facial nucleus at 8 weeks post-axotomy and nerve repair. The figure illustrates the expression levels of ChAT-positive cells in the facial nucleus across three groups: sham, axotomy, and the nerve repair groups (Forward Suture and Reverse Suture) at 8 weeks post-injury. The axotomy group exhibited a significant reduction in ChAT-positive cells, reflecting impaired cholinergic activity due to the injury. In contrast, both the forward suture and reverse suture groups demonstrated a significant increase in ChAT-positive cells compared to the axotomy group, suggesting partial restoration of cholinergic function facilitated by the nerve repair techniques. Data are expressed as means ± SEM. Statistical significance is indicated by (* *p* < 0.05 vs. Sham group) and (# *p* < 0.05 vs. Axotomy group). Scale bars: 200 μm.

We evaluated the expression levels of choline acetyltransferase (ChAT), a key enzyme involved in the synthesis of the neurotransmitter acetylcholine, within the facial nucleus at 8 weeks following facial nerve injury induced by axotomy. At 8 weeks post-injury, the axotomy group demonstrated a significant reduction in the number of ChAT-positive cells within the facial nucleus. This decrease is indicative of impaired cholinergic activity due to the nerve injury. In contrast, both the forward suture and reverse suture groups showed a marked increase in ChAT-positive cells compared to the axotomy group. This suggests a partial restoration of cholinergic function in these groups, likely facilitated by the nerve repair techniques employed. Quantitatively, statistical analysis revealed a significant difference among the groups (F = 19.162, *p* < 0.001) (Figure 2), highlighting the efficacy of the suturing methods in promoting nerve regeneration and functional recovery. Importantly, no significant difference in ChAT-positive cell number was observed between the forward suture and reverse suture groups (*p* > 0.05).

### 3.3. Expression of Iba-1 in the Facial Nucleus Following Facial Nerve Injury

**Figure 3:**
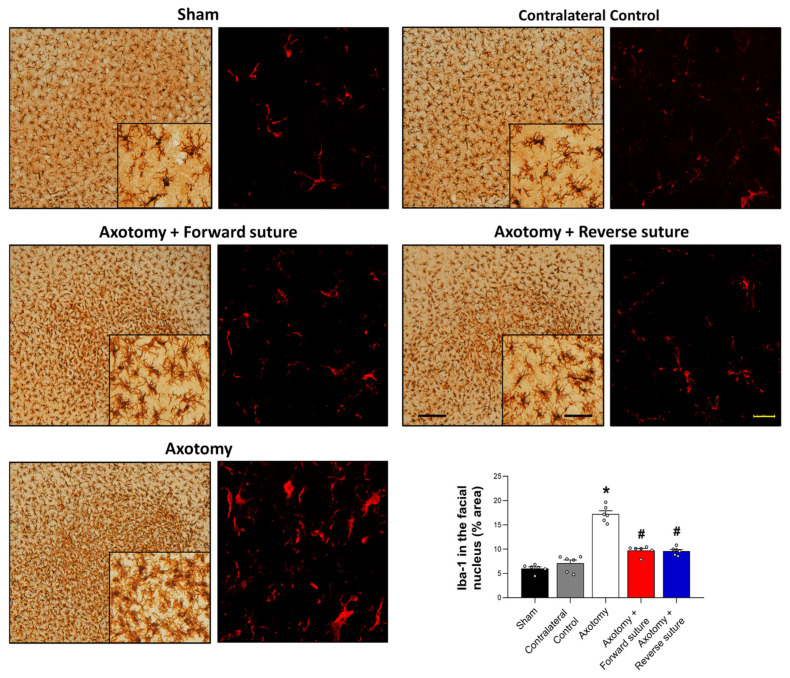
Expression of Iba-1 in the facial nucleus at 8 weeks post-facial nerve injury. The figure displays the expression levels of Iba-1, a marker for activated microglia, in the facial nucleus among different groups: sham, axotomy. The axotomy group showed a significant increase in Iba-1 expression, indicating elevated microglial activation due to the nerve injury. In contrast, both the forward suture and reverse suture groups exhibited reduced Iba-1 expression compared to the axotomy group, suggesting decreased microglial activity and mitigate the impact of nerve injury. Data are presented as means ± SEM. Statistical significance is denoted by (* *p* < 0.05 vs. Sham group) and (# *p* < 0.05 vs. Axotomy group). Scale bars: left, 200 μm; center, 50 μm; right, 20 μm.

We assessed microglial cell numbers in the facial nucleus at 8 weeks post-facial nerve injury, using ionized calcium-binding adaptor molecule 1 (Iba-1) as a marker for activated microglia. Following facial nerve axotomy, the axotomy group exhibited a significant increase in Iba-1 expression compared to the sham group, indicating heightened microglial activation in response to nerve injury. In contrast, both the forward suture and reverse suture groups showed a decrease in Iba-1 expression compared to the axotomy group. This suggests a reduction in microglial activity, potentially reflecting a more favorable environment for nerve regeneration due to the reparative effects of the suturing techniques. The statistical analysis confirmed a significant difference among the groups (F = 75.928, *p* < 0.001) (Figure 3), underscoring the potential of both suturing methods to attenuate microglial activation and promote neural repair. Importantly, no significant difference in Iba-1 expression was observed between the forward suture and reverse suture groups (*p* > 0.05).

### 3.4. Expression of SIRT1 in the Facial Nucleus Following Facial Nerve Injury

**Figure 4:**
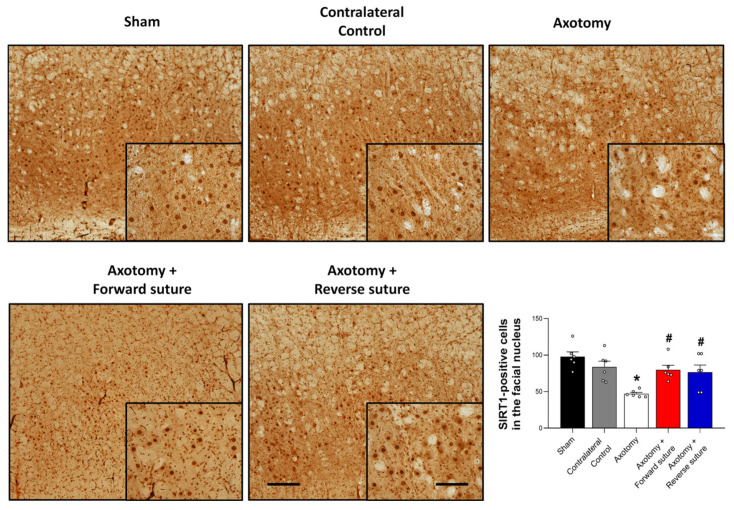
Expression of SIRT1 in the facial nucleus at 8 weeks following facial nerve injury. The figure shows decreased SIRT1-positive cells in the axotomy group compared to the sham group. Both the forward suture and reverse suture groups exhibit a significant increase in SIRT1-positive cells within the facial nucleus relative to the axotomy group. These results suggest that both suturing techniques enhance SIRT1 expression in response to facial nerve injury. Data are expressed as means ± SEM. Statistical significance is indicated by (* *p* < 0.05 vs. Sham group) and (# *p* < 0.05 vs. Axotomy group). Scale bars: left, 200 μm; right, 50 μm.

SIRT1 plays a key role in neuropathic pain caused by peripheral nerve injury by reducing oxidative stress and inflammation. Following facial nerve injury induced by axotomy, we observed changes in SIRT1 expression within the facial nucleus in the central nervous system. The axotomy group exhibited a decrease in SIRT1-positive cells in the facial nucleus compared to the sham group. However, both forward and reverse suturing techniques led to an increase in SIRT1-positive cells compared to the axotomy group (F = 7.184, *p* < 0.001) (Figure 4). These findings indicate that both suturing techniques enhance SIRT1 expression in response to facial nerve injury, suggesting a potential role for SIRT1-associated pathways in neuronal recovery and repair. Importantly, no significant difference in SIRT1-positive cell number was observed between the forward suture and reverse suture groups (*p* > 0.05).

## 4. Discussion

In this study, we investigated the recovery of motor and neural function following forward and reverse suturing after facial nerve axotomy. Our behavioral study results demonstrated that both suturing techniques significantly improved motor function recovery, as evidenced by enhanced whisker movement and eyelid blink reflex tests compared to the axotomy group. Following facial nerve axotomy, there was a decrease in the number of ChAT-positive and SIRT1-positive cells in the facial nucleus. However, both forward and reverse suturing methods ameliorated these cellular deficits. Additionally, facial nerve axotomy led to increased microglial activation, which was significantly reduced by both suturing techniques. These findings underscore the potential of forward and reverse suturing to enhance neural repair and functional recovery following facial nerve injury.

In our prior study using the same facial nerve axotomy and autograft model, we showed that forward and reverse suturing produced similar degrees of peripheral nerve regeneration and behavioral recovery, with no significant differences in axon counts or ultrastructural features between graft orientations. That study, however, did not examine how these surgical strategies influence the central facial nucleus. The present work addresses this gap by demonstrating that forward and reverse suturing also yield comparable patterns of central adaptation, characterized by partial restoration of ChAT- and SIRT1-positive neurons and attenuation of Iba-1-defined microglial activation within the facial nucleus. By integrating behavioral testing with central molecular readouts, our data indicate that the clinical equivalence of forward and reverse graft orientation extends beyond the peripheral nerve to the level of central motor circuitry, and they identify specific cholinergic, inflammatory, and SIRT1-related pathways that likely support functional recovery regardless of graft polarity.

Facial nerve axotomy results in permanent functional loss when not treated with appropriate surgical intervention. This condition leads to facial paralysis, which is characterized by weakened or completely lost facial movement [1]. In our study, facial nerve injury was induced through axotomy, and facial nerve function was assessed behaviorally using blink and whisker movement reflex tests. At 8 weeks post-axotomy, there was no recovery in facial nerve function based on these reflex tests. Additionally, persistent facial nerve injury was observed behaviorally at 12 weeks post-axotomy. Our previous research demonstrated improvements in behavioral function 4 weeks after forward and reverse suturing following facial nerve axotomy [6]. In the present study, significant improvements in motor function were observed 8 weeks after forward and reverse suturing treatments. Although complete axonal recovery was not achieved, peripheral facial nerve regeneration was improved compared to axotomy alone, as evidenced by toluidine blue staining and TEM analysis. These findings suggest that facial nerve injury due to axotomy may benefit from direct nerve graft suturing, which enhances the recovery of peripheral facial nerve axons and improves motor function.

Following axotomy, we confirmed that facial nerve regeneration and motor function recovery occurred through both forward and reverse suturing. In this study, a 5 mm gap created by facial nerve axotomy was bridged using an autologous nerve graft, and we confirmed that both forward and reverse suturing supported facial nerve regeneration and motor recovery [6]. In general, maintaining the original fascicular alignment of the graft in the same proximal-to-distal direction as the host nerve is considered important, because this orientation facilitates more precise axonal regeneration, enhances connectivity between the proximal and distal stumps, and minimizes axonal loss at the coaptation sites. Such alignment increases the specificity of regeneration and is thought to promote better functional outcomes, so forward suturing is often regarded as providing a more natural and effective pathway for nerve regeneration and motor function recovery [11]. However, reversing the polarity of nerve autografts can result in comparable levels of nerve function recovery, with no significant differences observed in regeneration outcomes between normal and reversed orientations [4]. Both reversed and non-reversed nerve grafts demonstrated similar conduction velocities and amplitudes, indicating that nerve function recovery was comparable regardless of graft orientation [5]. Similarly, after bilateral common peroneal nerve transection, there were no significant differences in motor conduction velocity, axon count, or density between forward and reverse nerve grafts [12]. In our previous studies, although axon numbers decreased up to 4 weeks post-suturing, partial recovery was observed by the 8th week, with no significant differences between the suturing directions [6]. Thus, in peripheral nerve injury induced by axotomy, there were no significant behavioral or histological differences, indicating that both forward and reverse suturing are effective methods for nerve regeneration.

Eight weeks post-facial nerve axotomy, there was a reduction in ChAT-positive cells within the facial nucleus of the central nervous system. ChAT, primarily produced in the facial nucleus, plays a critical role as an enzyme in the synthesis of acetylcholine, essential for motor neuron function. Thus, a reduction in ChAT levels may potentially impact motor function. In animal models of peripheral nerve injury, a sustained decrease in ChAT expression levels has been observed even up to 16 weeks after injury, which correlates with impaired motor recovery [13]. Similarly, our previous studies showed a decrease in ChAT-positive cells in the facial nucleus following facial nerve axotomy [14]. While ChAT expression temporarily decreased in cases of compressive injury, it typically recovered three weeks post-injury. However, after axotomy, ChAT expression was significantly reduced, and the number of ChAT-positive cells remained decreased even at 12 weeks post-injury. An incremental increase in ChAT-expressing neurons is associated with the recovery of facial motor function. In our results, compared to the axotomy group, both forward and reverse suturing groups showed an increase in ChAT levels in the facial nucleus at 8 weeks post-injury. Therefore, both suturing directions appear to improve motor neuron injury in the facial nucleus of the central nervous system.

Facial nerve injury caused by axotomy resulted in excessive microglial activation in the facial nucleus of the central nervous system [15]. Peripheral nerve injury is known to trigger microglial activation within the central nervous system, as evidenced by increased microglial proliferation and activation in response to motoneuronal injury. This response can be modulated by promoting motoneuron recovery, which subsequently reduces microglial activity [16]. Facial nerve axotomy can lead to the activation of Iba-1 in the facial nucleus of the central nervous system [14]. Increased neuroinflammation in the central nervous system, characterized by the persistent activation of microglia and the release of pro-inflammatory mediators, can affect motor neurons and potentially lead to impairments in motor function [17]. Overactive microglia can release neurotoxic substances, which may exacerbate neural damage and hinder recovery. Therefore, reducing neuroinflammation is essential for slowing the progression of neurodegenerative diseases. Strategies to regulate microglial activity are crucial for improving outcomes in nerve injuries and neurodegenerative conditions [18]. Both forward and reverse suturing techniques were found to reduce Iba-1 activation in the facial nucleus. Therefore, these suturing methods appear to mitigate microglial activation, thereby improving outcomes in facial nerve injury.

In our findings, SIRT1 expression in the facial nucleus decreased 8 weeks post-facial nerve injury. SIRT1 is known to play a critical role in peripheral nerve injuries, primarily through its ability to reduce oxidative stress and inflammation [19]. SIRT1 has been shown to modulate microglial activation and reduce neuroinflammation, which is essential for mitigating the progression of neurodegenerative diseases [20]. By deacetylating key transcription factors, SIRT1 can inhibit the expression of pro-inflammatory cytokines and promote the survival of neurons. Recent studies indicate that enhancing SIRT1 activity could significantly improve recovery outcomes in nerve injuries. This improvement is attributed to SIRT1’s ability to modulate the inflammatory response and protect neuronal integrity, thereby promoting a more conducive environment for nerve repair and regeneration [21]. Facial nerve injury induced by axotomy resulted in a decrease in the number of SIRT1-positive cells in the facial nucleus of the central nervous system. Both forward and reverse suturing techniques were effective in increasing SIRT1 expression compared to the untreated injury group. This pattern suggests that increased SIRT1 expression may contribute to a more favorable environment for neuronal recovery and repair, and raises the possibility that targeting SIRT1 could be explored as a potential therapeutic strategy in facial nerve injuries; however, the present data are correlative and do not establish a causal role.

Several limitations of this study should be acknowledged. First, the sample size was relatively small (*n* = 6 per group), and all experiments were conducted in young male Sprague–Dawley rats, which may limit the generalizability of our findings to other ages, sexes, species, and to clinical populations. Second, we did not perform electrophysiological assessments (e.g., nerve conduction studies or electromyography), so the functional interpretation relies on behavioral testing in combination with histological and immunohistochemical readouts. Third, although we observed parallel improvements in behavioral outcomes and central markers (ChAT, Iba-1, and SIRT1), we did not perform formal correlation analyses between individual functional scores and histological measures; therefore, the strength of the relationship between these domains remains unclear. Fourth, the observed changes in ChAT and SIRT1 within the facial nucleus are correlational. We did not manipulate these pathways directly (e.g., pharmacological inhibition/activation of SIRT1 or targeted modulation of cholinergic signaling), and therefore cannot conclude that these molecules are causally required for the observed motor recovery. Future studies employing SIRT1 agonists/antagonists or genetic approaches, as well as interventions that directly alter ChAT activity, combined with electrophysiological measurements and quantitative correlation analyses, will be necessary to establish causal links between these pathways, tissue-level changes, and functional outcomes after facial nerve repair.

## 5. Conclusions

Collectively, these findings extend our previous peripheral nerve-level observations and provide a mechanistic central nervous system framework explaining why forward and reverse autologous grafts achieve comparable functional outcomes after facial nerve repair. In this rat facial nerve axotomy model, both forward and reverse autologous suturing techniques enhanced motor function recovery and produced similar behavioral outcomes, and at the level of the facial nucleus they were associated with partial restoration of ChAT- and SIRT1-positive neurons and reduced Iba-1-defined microglial activation, indicating broadly similar central adaptations that may support neural repair. Notably, the upregulation of ChAT and SIRT1 expression in the suturing groups is associated with a more favorable regenerative environment within the facial nucleus and may reflect molecular adaptations that support neural repair, although these relationships are correlative and do not establish causality.

## Data Availability

The original contributions presented in this study are included in the article. Further inquiries can be directed to the corresponding author.
